# A systematic review of interventions in primary care to improve health literacy for chronic disease behavioral risk factors

**DOI:** 10.1186/1471-2296-13-49

**Published:** 2012-06-01

**Authors:** Jane Taggart, Anna Williams, Sarah Dennis, Anthony Newall, Tim Shortus, Nicholas Zwar, Elizabeth Denney-Wilson, Mark F Harris

**Affiliations:** 1 Centre for Primary Health Care and Equity, University of New South Wales, Sydney, 2052, Australia; 2 School of Public Health and Community Medicine, University of New South Wales, Sydney, 2052, Australia

**Keywords:** Health literacy, Behavioral risk factors

## Abstract

**Background:**

To evaluate the effectiveness of interventions used in primary care to improve health literacy for change in smoking, nutrition, alcohol, physical activity and weight (SNAPW).

**Methods:**

A systematic review of intervention studies that included outcomes for health literacy and SNAPW behavioral risk behaviors implemented in primary care settings.

We searched the Cochrane Library, Johanna Briggs Institute, Medline, Embase, CINAHL, Psychinfo, Web of Science, Scopus, APAIS, Australasian Medical Index, Google Scholar, Community of Science and four targeted journals (Patient Education and Counseling, Health Education and Behaviour, American Journal of Preventive Medicine and Preventive Medicine).

Study inclusion criteria: Adults over 18 years; undertaken in a primary care setting within an Organisation for Economic Co-operation and Development (OECD) country; interventions with at least one measure of health literacy and promoting positive change in smoking, nutrition, alcohol, physical activity and/or weight; measure at least one outcome associated with health literacy and report a SNAPW outcome; and experimental and quasi-experimental studies, cohort, observational and controlled and non-controlled before and after studies.

Papers were assessed and screened by two researchers (JT, AW) and uncertain or excluded studies were reviewed by a third researcher (MH). Data were extracted from the included studies by two researchers (JT, AW). Effectiveness studies were quality assessed. A typology of interventions was thematically derived from the studies by grouping the SNAPW interventions into six broad categories: individual motivational interviewing and counseling; group education; multiple interventions (combination of interventions); written materials; telephone coaching or counseling; and computer or web based interventions. Interventions were classified by intensity of contact with the subjects (High ≥ 8 points of contact/hours; Moderate >3 and <8; Low ≤ 3 points of contact hours) and setting (primary health, community or other).

Studies were analyzed by intervention category and whether significant positive changes in SNAPW and health literacy outcomes were reported.

**Results:**

52 studies were included. Many different intervention types and settings were associated with change in health literacy (73% of all studies) and change in SNAPW (75% of studies). More low intensity interventions reported significant positive outcomes for SNAPW (43% of studies) compared with high intensity interventions (33% of studies). More interventions in primary health care than the community were effective in supporting smoking cessation whereas the reverse was true for diet and physical activity interventions.

**Conclusion:**

Group and individual interventions of varying intensity in primary health care and community settings are useful in supporting sustained change in health literacy for change in behavioral risk factors. Certain aspects of risk behavior may be better handled in clinical settings while others more effectively in the community. Our findings have implications for the design of programs.

## Background

The decisions and actions which people make about their lifestyle behaviour are effected by their level of health literacy [1] and are key influences on the prevention and management of chronic illness [2,3].

There is a strong association between the SNAPW risk factors (smoking, poor nutrition, hazardous or harmful use of alcohol, inadequate physical activity and overweight and obesity) and chronic non communicable diseases such as diabetes and cardiovascular disease [4] and these risk factors are major contributors to the burden of chronic disease in Australia [5] and internationally [6,7].

Health literacy, as defined by Nutbeam [8], has three levels: Functional health literacy that includes the basic reading and writing skills needed to be able to function in daily life; communicative or interactive health literacy that includes more advanced cognitive and literacy skills which combine with social skills to enable someone to participate in a range of activities and apply information to changing situations; and critical health literacy that comprises of more advanced cognitive and social skills that a person can use to exert more control over their lives.

People with low health literacy levels have poorer health outcomes than those with higher levels of health literacy [2,3,9]. They have less knowledge of diseases and self care [10]; worse self management skills [11]; lower uptake of screening [12,13]; lower medication compliance rates [14]; and higher rates of hospitalisation [3]. People with low health literacy also have lower levels of engagement in health promoting behaviours and are more likely to smoke especially in adolescence and as young adults [3]. By contrast the benefits of high levels of health literacy include improved preventive care and early detection of illness and disease, ability to access the most appropriate form of health care and management of chronic disease [15]. In a survey conducted in the United Kingdom higher levels of health literacy were associated with specific health behaviours including the likelihood of eating at least five portions of fruit and vegetables a day or being a nonsmoker independently of age, education, gender, ethnicity and income [1].

A range of simple and complex interventions have been used to improve the health of people with low levels of literacy. For example, group education within a disadvantaged community, written materials and resources with simplified language and pictures, and individual counselling through primary health care. Two systematic reviews of interventions for improving the health of people with low literacy have been published [16,17]. The first reviewed complex interventions to improve the health of people with limited literacy and reported that a variety of complex interventions can improve some health related outcomes, particularly health knowledge and self-efficacy. The second reported mixed results for interventions targeting people with low literacy and that limitations in study design make it difficult to draw conclusions. Both these reviews included studies targeting any health condition and conducted in any setting. We conducted a systematic review to evaluate the effectiveness of interventions used specifically in primary health care to improve health literacy in adults to support change in smoking, nutrition, alcohol, physical activity and weight (SNAPW) behaviour.

The research question is: Which interventions have been found to be effective in changing health literacy and the SNAPW lifestyle risk factors?

## Methods

This review was informed by input from international, national and state health policy practitioners, academics and clinicians to ensure relevance to current health initiatives and priorities through establishment of an international reference group and semi-structured interviews with targeted “experts” related to the topic in shaping the research questions and sourcing literature.

### Conceptual framework

**Figure 1:**
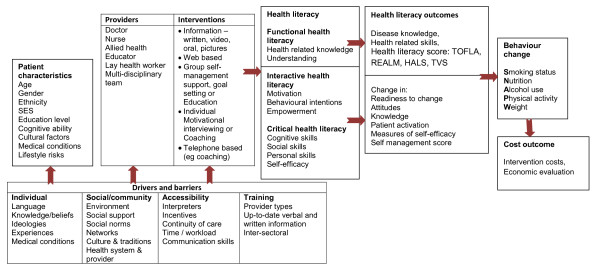
Conceptual framework for the review.

A conceptual framework was developed to guide the review by bringing together the three levels of health literacy with patient characteristics, providers and interventions, the drivers and barriers and possible outcome measures of behavioural change for SNAPW (Figure 1).

### Search strategy

The search strategy targeted peer reviewed, published and non-published literature. Only published intervention studies are reported in this paper.

We searched databases including the Cochrane Library, Johanna Briggs Institute, Medline, Embase, CINAHL, Psychinfo, Web of Science, Scopus, APAIS, Australasian Medical Index, Google Scholar, Community of Science and four targeted journals (Patient Education and Counseling, Health Education and Behaviour, American Journal of Preventive Medicine and Preventive Medicine). Search strategies were developed for each database with limits for English language, published between January 1^st^ 1985 and April 30^th^ 2009 and adults from the age of 18 years or above. A filter for primary health care was applied. An example of one of the search strategies is shown in Table 1.

**Table 1:** Medline search strategy

**Search Fields**	**Database specific terms (Text& MESH)**
**Health**	Patient Education as Topic/or exp Health Education/or health literacy.mp. or exp
**Literacy**	Health Knowledge, Attitudes, Practice/exp Patient Compliance/exp Educational Status/(functional adj health adj literacy).tw. interactive health literacy.tw. critical health literacy.tw.
**Outcomes**	wrat.tw. realm.tw. tofhla.tw. hals.tw. social support scale.tw. diabetes care profile.tw. newest vital sign.tw. exp PhysicianPatient Relations/exp Self Efficacy/exp rating scale/or exp scoring system/exp questionnaire/ exp Psychological Rating Scale/
**Primary**	Primary Health Care/exp Comprehensive Health Care/exp Patient Care
**Health Care**	Management/exp Family Practice/exp Physicians, Family/exp Community Health Services/ (primary adj1 (care or health)).tw. (family adj1 (doct$ or medic$ or pract$ or physic$)).tw. (general adj1 pract$).tw. (gp or gps).tw.
**Interventions**	exp Health Promotion/exp Motivation/ motivation$ interviewing.tw. exp Behavior Therapy/exp Risk Reduction Behavior/exp Consumer Health Information/exp Smoking Cessation/self management.mp. exercise.mp. or exp Exercise/brief intervention.mp. exp nutrition assessment/exp Patient Education as Topic/exp Self Care/ed [Education] exp Self Care/ "group education".mp. exp Education/
**Lifestyle risk factors**	exp Smoking/ec, pc [Economics, Prevention & Control] exp drinking behavior/or exp alcohol drinking/or exp feeding behavior/or exp habits/or exp health behavior/ exp Exercise/exp Overweight/exp Obesity/exp risk factors/exp Life Style/exp Health Behavior/

### Inclusion criteria

#### Participants

Adults aged 18 years and over. ***Setting*** To be included, studies had to be undertaken in a primary care setting within an Organisation for Economic Co-operation and Development (OECD) country. Primary care is first level care provided by a suitably trained workforce and supported by integrated referral systems. We included studies if the interventions were delivered by: family practices, local primary care organisations, generalist community health services (including home nursing, school health, child & family health, counseling, allied health, continence, aboriginal health, multicultural health & language services, health education/information, information, advocacy & support, home care & support services, transport services).

We excluded studies where the interventions were delivered by specialist services based in the community. These services included: HIV/AIDS, including multicultural & allied health, mental health, Aged Care Assessment Teams, respite care, alcohol tobacco and other drugs, treatment, rehabilitation, child protection, special interest non government organizations, technical aids for disabled, specialist and hospital based services [18].

#### Intervention

Interventions had to include at least one measure of health literacy and promote positive change in lifestyle behaviours for smoking, nutrition, alcohol, physical activity and/or weight.

We used Nutbeam’s definition of three levels of health literacy [8] including functional health literacy, communicative or interactive health literacy and critical health literacy.

#### Outcomes

Studies had to measure at least one outcome associated with health literacy and report a SNAPW outcome.

We identified a number of health literacy outcome scales and measures for the review. For functional health literacy these included the Wide Range Achievement Test (WRAT), Rapid Estimate of Adult Literacy in Medicine (REALM), Test of Functional Health Literacy in Adults (TFHLA), Health Activity Literacy Scale, Newest Vital Sign, Short Assessment for Spanish Speaking Adults and the disease specific knowledge assessment, the Diabetes Care Profile. We could not identify established tools for measuring interactive and critical health literacy so we looked to the self management literature for instruments that measure the concepts of self-efficacy, patient motivation, confidence and broader social support such as the Diabetes Self Efficacy Scale, the Social Support Survey and measures of Prochaska and DiClemente's Stages of Change Model [19].

Interventions were assessed to be effective if a statistically significant positive change was reported for health literacy or a SNAPW outcome.

#### Study design

Experimental and quasi-experimental studies, cohort, observational and controlled and non-controlled before and after studies were included.

### Assessment and screening

The assessment and screening process was undertaken by two researchers (JT, AW) who assessed half the retrieved studies independently. Uncertain or excluded studies were reviewed by a third researcher (MH) and discussed by the team where any uncertainties remained. Included studies were screened by title and abstract and the full paper verified by assessing the contents of the paper based on the set criteria. Data were extracted from the included studies by two researchers (JT, AW) including general information about the studies, intervention descriptions, health literacy and SNAPW measures, health literacy and SNAPW outcomes and conclusions by authors. All excluded studies were checked by a member of the team. Effectiveness studies were quality assessed by one researcher (SD) using a published quality checklist [20,21]. A second researcher performed quality assessment on a 20% overlapping random sample of the studies for verification (AW). The combined assessment produced a Kappa score of 0.72.

### Developing a typology of interventions

A typology of interventions was thematically derived from the studies by one researcher (AW) and verified by a second researcher (JT) by grouping the SNAPW interventions into six broad categories: individual motivational interviewing and counseling; group education; multiple interventions (combination of interventions); written materials; telephone coaching or counseling; and computer or web based interventions. Each intervention was then classified by intensity of contact with the subjects (High ≥ 8 points of contact/hours; Moderate >3 and <8; Low ≤ 3 points of contact hours) and setting: primary health care, community or other. Primary health care services for example included general practice, Health Maintenance Organisations, community health and primary care clinics. Community included churches, neighbourhood coalitions and municipalities while other settings included hospital outpatients.

### Effectiveness of the interventions

Studies were analyzed by intervention category and whether significant positive changes in SNAPW and health literacy outcomes were reported. Analysis was undertaken for the significant outcomes for each intervention group. Quality scores and intensity ratings were described for significant findings.

## Results

**Figure 2:**
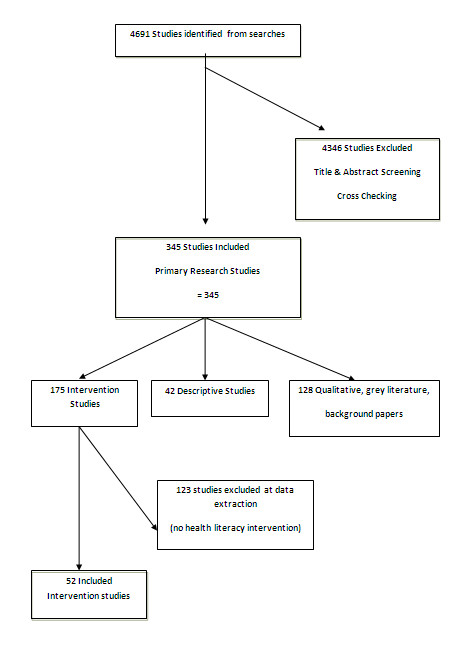
Flowchart of review.

We identified 52 intervention studies that were implemented in primary health care (n = 28), the community (n = 20) or other settings (n = 4) such as hospital outpatients clinic or worksite. Studies were from the US (n = 30), Australia/New Zealand (n = 4) and other OECD countries (n = 18). There were 29 randomized controlled trials (2 were clustered), 14 randomized trials, 6 before and after studies, 2 quasi experimental and a non-randomized controlled trial. See Figure 2 for review flowchart.

Group education was the most common intervention (n = 15), and nutrition (n = 34) and physical activity (n = 32) the most common SNAPW risk factors targeted. Only two studies targeted alcohol and neither demonstrated a change following the intervention (Table 2). Overall, 38 studies (73%) reported significant positive change in a health literacy outcome. Interventions of all types were associated with change in health literacy (Table 2). The majority of studies measured changes in participants’ stage of change (60%) compared to other measures of health literacy. Of these studies, 74% reported a statistically significant improvement in participant’s stage of change. Twenty eight (54%) reported significant outcomes for both health literacy and SNAPW. No significant positive health literacy or SNAPW outcomes were reported in 4 studies (Table 3). No studies reported significant negative results.

**Table 2:** Studies by intervention type and change in SNAPW and health literacy (figures are numbers of studies)

**Intervention type (no. of studies)**	**Intensity#**			SNAPW* outcome (No. sig. studies/No. studies measuring SNAPW) (%)						**Health literacy outcome (No. studies sig. outcome/No. studies measuring health literacy component) (%)**							
	**L**	**M**	**H**	**S**	**N**	**A**	**P**	**W**	**All SNAPW results (%)**	**Knowledge**	**Skills**	**Self efficacy**	**Stage change**	**Other health literacy**	**All health literacy results**		
Group education (15)	1	3	11	0/2	6/13	0/1	6/10	2/2	11/15 (73)	6/7	0	6/12	4/5	2/4	13/15 (87)
Individual counseling (11)	8	2	1	3/4	0/3	0	5/7	0/1	7/11 (64)	1/2	1/1	1/4	6/8	0/5	8/11 (73)
Multiple interventions (10)	1	5	4	1/3	5/7	0/1	2/6	2/3	9/10 (90)	3/6	0	2/4	5/6	0/1	7/10 (70)
Web/Computer (2)	2	0	0	0/1	2/2	0	1/1	0	2/2 (100)	0	0	0	1/2	0	1/2 (50)
Telephone (2)	0	1	1	0	0/1	0	0/1	0	0/2 (0)	1/1	0	1/1	1/1	0	2/2 (100)
Written material (12)	10	2	0	4/6	7/8	0	4/6	0/1	10/12 (83)	1/1	0/1	2/3	6/9	1/2	7/12 (58)
TOTALS (52)	22	13	17	8/16	20/34	0/2	18/32	4/7	39/52 (75)	11/17	1/2	12/24	23/31	3/12	38/52 (73)

#L = Low, M = Medium, H = High; * S = Smoking, N = Nutrition, A = Alcohol consumption, P = Physical activity, W = Weight; ^i^ Includes social support, attitudes, beliefs, awareness and more likely to read information.

**Table 3:** Study characteristics by outcomes in health literacy and SNAPW (figures are number of studies)

	Intensity* (n)			**Follow-up time (months)**			**Setting (n)**			**Quality (n)**		
Significant outcome for:	L	M	H	<6	6–12	>12	PHC(29)	Com (21)	Other(4)	L	M	H
Health Literacy (38)	15	8	15	13	20^#^	5	20	15	3	4	26	8
SNAPW (39)	17	9	13	13	19^#^	6	21	16	2	4	27	8
Both HL and SNAPW (28)	13	4	11	9	3^	6	15	12	1	13	5	10
No sig. outcome (4)	2	2	0	1	2	1	3	1	0	1	2	1

*Intensity rating: High - 8 or more hours/points of contact for patient, Moderate - >3 and < 8 hours/points of contact for patient, Low <3 or less hours/points of contact for patient; ^#^ 9 had followup of 52 weeks; ^ 5 had follow-up of 52 weeks.

Overall, 39 (75%) studies reported a change in one or more SNAPW risk factors. Telephone counseling was the only intervention not associated with positive significant change in SNAPW behaviors. Individual counseling and written materials were more effective in achieving impacts around smoking cessation compared to group education. All intervention types were similarly effective for physical activity while written materials and multiple interventions were the most effective at positively changing nutrition. Table 4 gives a brief description of the interventions that were successful in changing health literacy and/or SNAPW.

**Table 4:** Effective interventions for health literacy

**Effective interventions**	**Participants**	**Setting**	**Quality^**	**RCT**	**SNAPW**^**#**^
GROUP EDUCATION					
4 to 5 group empowerment sessions over 7 months [22]	Patients with diabetes from 7 primary care centres	Community health	H	RCT	
*40 hour group education session over 4 weeks with participants following preset dietary goals [23]	Mostly white American	Other (Centre of Excellence)	H	RCT	N, P, W
Church-based program tailored and culturally relevant that included awareness raising activities and exercise and cooking classes over 2 years [24]	Samoan and Tongan	Community	H		W
*Language specific self management program of 2.5 hour weekly sessions for 6 weeks with audiocassette and booklet [25]	Greek, Vietnamese, Chinese and Italian	Community	M	RCT	P
Culturally sensitive curriculum in small and large groups and support over 10 months [26]	Mexican American/Latina women of low socio-economic, low education	Community	M		P
2.5 day program then weekly group education over 6 months and small group support [27]	Mostly Caucasian	Primary Care clinic	M	RCT	N, P
*Chronic disease self management group program of 15 hours over 6 weeks [28]	Mostly Mexican born, low socio-economic, low education	Community	M		N, P
*Small groups that met for an hour one night a week for 16 weeks and then every second week for a further 8 weeks [29]	Mean age 46 yrs	Community	M	RCT	P
Monthly group meetings over 6 months and an additional individual session if requested by patient or needed [30]	Mostly white American	Primary care	M		N
Classes and follow-up phone calls over 1 year [31]	Women 20 to 50 yrs	Community	M		
*10 weekly group education sessions [32]	Mean age around 73	Hospital outpatient	M	RCT	
*6 × 2 hour classes targeting stage of change and culturally appropriate resources and decision tree with periodic group support meetings after the class series [33]	Mostly Latino then African American, low socio-economic, low education	Community	M		
3 × 2 hr Prochaska-based stage matched group education sessions [34]	Low socio-economic and education	Primary care	M	RCT	N
WRITTEN MATERIALS					
*Computer generated tailored nutrition newsletters & profile feedback related to stage of change [35]	Majority African Americans	General practice	H	RCT	N
3 iterative letters [36]	Educated, mean age 49 yrs	Community	M	RCT	N, P
*3 repeated mailings of self help manuals and motivational messages related to stage of change [37]	Mostly Caucasian	Community	M		P
1 tailored or non-tailored letter [38]	Smokers aged 17 to 65 yrs	General practice	M	RCT	S
*12 week mailed lifestyle intervention program [39]	Primarily Caucasian women	Community	L		P
3 computer generated reports based on stage of change for each risk factor [40]	Mostly Caucasian	Primary care	M	RCT	S, N
3 computer generated reports based on stage of change for each risk factor [41]	Mostly Caucasian	Community	M	RCT	S, N
INDIVIDUAL COUNSELING					
Lifestyle counseling by a doctor with video and written materials [42]	Mean age about 53 yrs	Primary health care	H		
Exercise prescription provided by GP, 1 counseling session with nurse and materials [43]	Mean age 59 yrs	Primary health care	H		P
1–3 individual brief counseling by a nurse [44]	Low socio-economic, low education	Primary health care	M	RCT	P
One individual consultation by a nurse [45]	Practice nurses and their patients	Primary health care	M		
One individual counseling by a registrar [46]	Mean age 41 yrs	Primary health care	M		
*One motivational counseling and patient setting targets [47]	Mostly female	Primary health care	M	RCT	P
*Two individual counseling sessions by a physician and two follow-up phone calls [48]	Hypertension and/or hypercholesterolemia and/or non insulin dependent diabetes	General practice	M	RCT	P
*12 to 20 week individual counseling for COPD patients [49]	Scandinavian	Primary health care	L	RCT	S, P
MULTIPLE INTERVENTIONS					
6 or 7 × 60min classes and multiple mail/telephone follow-up calls (Stanford Nutrition Action Program) [50]	Mostly Hispanic born in the US, poor, low education and literacy	Community	H	RCT	N
*1 mailing of stage based booklets with provider endorsement and 2 motivational phone counseling sessions [51]	Majority Caucasian	General practice	M	RCT	N
*Interactive computer sessions with feedback from a nurse, a risk factor manual, brief audio tapes, stress management and exercise instructions [52]	Mostly African American	Primary health care	M		S
Group education sessions with individual counseling [53]	47% high school education or greater	General practice	M	RCT	W
Various interventions designed by neighbourhood coalitions that have GP representation [54]	Low socio-economic, low education	Community	M		N
Stages of change based and counseling and written materials provided by a nurse [55]	Mostly female (70%) mean age 42.4 yrs	General practice	L		P
Range of health promotion activities by lay community members [56]	Japanese. Age range 30 to 59 yrs	Community	M		N, P
TELEPHONE					
Two individual education sessions over the phone plus a mailed brochure [57]	Mostly middle aged, married, Non Hispanic black men	Community	H	RCT	
6 months telephone counseling and exercise logs [58]	Well educated Caucasian	Community	L		
COMPUTER					
*Self guided interactive program with 2 reminder phone calls [59]	Low socio economic, African and white American women	Community	M		N

^Quality of study H = High, M = Medium, L = Low; ^#^ SNAPW significant positive outcome reported, S = Smoking, N = Nutrition, A = Alcohol, P = Physical activity, W = Weight; *Follow-up < 6 months.

Interventions were of variable intensity with slightly more studies evaluating low intensity interventions (43%) reporting significant positive outcomes for SNAPW risk factors compared with those evaluating high intensity interventions (33%). The same number of low and high intensity interventions reported significant positive outcomes for health literacy (39% each).

Of the high quality studies reporting significant positive outcomes for health literacy (8 studies) [22-24,35,42,43,50,57], three included a group education intervention, two individual counseling, one written, one telephone and a multiple intervention (group education with mail/telephone follow-up). Five of these 8 studies reported change in at least one behavioral risk factor but only one reported significant positive change at 12 months or greater (in weight but not physical activity or nutrition). This was a culturally tailored church based group education program over two years that included awareness raising activities and exercise and cooking classes [24].

Thirteen (86%) studies evaluating group education interventions reported significant positive outcomes for health literacy, 11 (73%) for lifestyle behavior with nine (60%) of these reporting significant positive outcomes in both health literacy and lifestyle behavior.

There were four group education interventions that were successful in positively changing health literacy but not lifestyle behavior [22,31-33]. Two of these measured outcomes at 12 months. These included classes and follow-up phone calls over one year (did not change smoking, nutrition or physical activity) [31] and four to five group empowerment sessions over 7 months (did not change nutrition) [22]. The other two studies had short follow up periods of less than 20 weeks.

Most individual counseling interventions were brief with eight (73%) reporting significant improvements in health literacy [42-49]. Four of the eight studies reporting improvements in health literacy consisted of one counseling session [43,45-47] and two of these also demonstrated positive change in lifestyle behavior (smoking [46] and physical activity [47]). One of these individual counseling interventions included a physical activity prescription and follow-up call but reported no significant changes in physical activity [43]. Brief advice by a doctor followed by extensive counseling by a nurse changed smoking but not health literacy [60] and a lifestyle counseling program with video and written materials provided by a doctor changed health literacy but not physical activity [42].

Seven (58%) interventions using written materials reported significant outcomes in both health literacy and at least one lifestyle behavior. These seven interventions varied in intensity from one time mail outs to a 12 week mailed lifestyle program. There were significant positive outcomes for nutrition [35,40,41,53,61], smoking [38,40,41], and physical activity [37,53,61] The two telephone intervention studies reported significant positive outcomes in health literacy but no change in lifestyle risk factors [57,58].

Both computer interventions were of low intensity with short follow-up (5 and 8 weeks). One reported significant positive change in nutrition and physical activity but not smoking or health literacy [62] and the other positive results for health literacy and nutrition [59].

Multiple interventions used a mix of intervention types and intensities. Seven demonstrated significant outcomes in both health literacy and SNAPW (70%). Two of these included a telephone component as part of the intervention (telephone counseling and mailing stage-based booklets [51] and multiple mail and follow-up phone calls after group education classes [50]). Two interventions including group education sessions with individual counseling [53] and a diary [61] had significant positive outcomes for weight but not for nutrition and physical activity. Interventions implemented in primary health care settings (including general practice, primary care, health maintenance organizations and community health) were more successful at demonstrating change in smoking compared with interventions in community settings (50% success compared with 20%). Interventions in community settings were more likely to report positive change in physical activity (62% compared with 47%) and nutrition (65% compared with 56%). Both settings showed similar results for weight (both 50%) and health literacy (78% versus 75% for community settings). All individual counseling interventions were implemented in a primary health care setting.

## Discussion

Understanding and measuring patients’ health literacy in relation to behavioural risk factors is an important goal in the prevention and detection of chronic disease. It was therefore surprising to find relatively few studies measuring functional health literacy or components of interactive and critical health literacy (i.e. health knowledge, self-efficacy, patient motivation, confidence and social support) searched in this study. Our review supports the need to develop and validate better instruments for measuring health literacy (particularly interactive and critical health literacy) and for more studies to evaluate health literacy as an intermediate outcome rather than simply the health behavior as the endpoint. Since our review the Health Literacy Skills Instrument has been developed and validated to measure a persons ability to obtain and use health information using a skills-based approach [63]. This may be promising for future research to better understand health literacy and change in risk behaviors. Both group and individual interventions in primary health care and community settings demonstrated improved health literacy for change in behavioural risk factors. While health literacy results across the different settings were similar there was some variation in the results for SNAPW risk behaviours. Primary health care based interventions may be more effective with smoking cessation while interventions in the community setting may be more effective in changing nutrition and physical activity. This has implications for developing programs to reduce SNAPW risk factors. Health literacy for certain risk behaviours may be better suited to interventions based in clinical settings while others may be more effective in community settings. The reason that there were more effective intervention studies focusing on tobacco cessation in primary health care than community settings may be related to the increased availability of pharmacotherapy in primary health care.

No one intervention type appeared to be the most effective in increasing health literacy but there were some differences in their effectiveness with individual SNAPW risk behaviours. This has implications in the delivery of interventions to improve health literacy as well as specific SNAPW risk behaviours. For example, individual counselling and written materials may be a more effective way of improving health literacy for smoking cessation while multiple interventions and written materials may be more effective at improving health literacy for nutrition. Changing nutrition may require the highest level of health literacy compared with smoking as it requires knowledge and skills about how to improve one’s diet. This may be a greater challenge than knowing why one should not smoke and how to cease smoking.

The likelihood of interventions being effective did not appear to be related to the intensity of the intervention. A number of studies that evaluated lower intensity interventions (such as the use of written materials tailored to the stage of change) were effective in changing both health literacy and behavioral outcomes. Some of the low intensity interventions where subjects had ≤3 hours of contact or 3 points of contact were as successful in achieving significant outcomes in health literacy and SNAPW as some of the high intensity interventions where subjects had more than 8 hours or points of contact. This may not be the case for smoking cessation interventions using individual counseling. Systematic reviews on smoking cessation report interventions are more effective as the amount of contact time increases from less than 3 minutes to greater than 10 minutes [64] and if the intervention is conducted over four to seven sessions [65]. This is an important finding for policy and practice and will influence the calculation about benefits versus costs of interventions to be adopted and supported more widely within health systems. Targeting health literacy does not necessarily have to involve the implementation of extensive and potentially expensive interventions that might also require greater commitment (training, capacity) by clinicians implementing them.

Effective interventions may target multiple behaviors (such as both physical activity and diet) without compromising their effectiveness. However, simply combining multiple interventions into a large complex program without a coherent framework may not be effective. More research is required to establish the various combinations of interventions and their impact on health literacy for SNAPW and the associated capacity requirements. Further research is also required on health literacy for reducing alcohol consumption, web/computer type interventions and telephone interventions as few health literacy studies evaluating these were identified in this review. The results of this review need to be interpreted carefully, as the focus of the studies was often on behavioral risk factors rather than health literacy and studies used different measures for health literacy. We used a broad definition for health literacy which led to the inclusion of studies using proxy measures such as readiness for change, self efficacy and attitudes as specific instruments to measure interactive and critical health literacy could not be found. Health literacy was poorly indexed which resulted in searches being highly sensitive with poor specificity. Five of the studies included in the review were of low quality, however excluding them made little difference to the overall findings either in relation to health literacy or behavioral outcomes.

Another limitation is that the results show counts of studies with significant positive findings. This does not take into consideration sample size which could impact on the significance of a result. We found no studies with significant negative results which may be due to publication bias. Studies with non-significant findings may be less likely to be published. These results cannot be generalised to countries outside the OECD.

## Conclusions

Health literacy enables people to build their knowledge, skills and potential to make positive behaviour changes. Improving health literacy is more likely to lead to sustained behaviour change given that lower levels of health literacy are associated with poorer health outcomes. This review suggests that group and individual interventions of varying intensity in both primary health care and community settings may all be useful in supporting sustained change in health literacy for change in behavioural risk factors. There may be scope for some tailoring of the site and type of interventions depending on which risk factor is the focus. Our findings have implications for the design of programs, as less intense interventions may be as effective as more intensive ones. There is a need for more research to evaluate which interventions are best suited to developing health literacy for individual behaviours especially in disadvantaged populations.

## Abbreviations

SNAPW, Smoking, nutrition, alcohol, physical activity, weight; OECD, Organisation for Economic Co-operation and Development.

## Competing interests

The authors declare that they have no competing interests.

## Authors’ contributions

JT participated in the development and implementation of the methodology, the analysis and interpretation of results and was responsible for preparing the manuscript. AW participated in the development and implementation of the methodology, the analysis and interpretation of results and revising the manuscript. SD participated in the development and implementation of the methodology, the analysis and interpretation of results and revising the manuscript. AN participated in the development of the search strategy, interpretation of results and revising the manuscript. TS participated in the development of the methodology, completing the semi-structured interviews with the targeted “experts” in shaping the research questions, assisted in sourcing literature and in revising the manuscript. EDW participated in the development of the methodology, revision of the results and revising the manuscript. NZ participated in the development of the study and methodology and revision of results. MH was the Principal Investigator and contributed substantially to the development and implementation of the review, the interpretation of results and revising the manuscript. All authors read and approved the final manuscript.

## Pre-publication history

The pre-publication history for this paper can be accessed here:

http://www.biomedcentral.com/1471-2296/13/49/prepub
